# High ω3-polyunsaturated fatty acids in fat-1 mice prevent streptozotocin-induced Purkinje cell degeneration through BDNF-mediated autophagy

**DOI:** 10.1038/srep15465

**Published:** 2015-10-27

**Authors:** Dong Ho Bak, Enji Zhang, Min-Hee Yi, Do-Kyung Kim, Kyu Lim, Jwa-Jin Kim, Dong Woon Kim

**Affiliations:** 1 Department of Anatomy, College of Medicine, Konyang University of Korea, Daejeon, South Korea; 2 Department of Pharmacology, College of Medicine, Konyang University of Korea, Daejeon, South Korea; 3 Department of Anatomy, Brain Research Institute, Chungnam National University School of Medicine, Daejeon, South Korea; 4 Department of Biochemistry, Infection Signaling Network Research Center, Chungnam National University School of Medicine, Daejeon, South Korea; 5 Department of Anesthesiology, Yanbian University Hospital, Yanbian, 133000, China

## Abstract

Loss of Purkinje cells has been implicated in the development of diabetic neuropathy, and this degeneration is characterized by impairment of autophagic processes. We evaluated whether fat-1 transgenic mice, a well-established animal model that endogenously synthesizes ω3 polyunsaturated fatty acids (ω3-PUFA), are protected from Purkinje cell degeneration in streptozotocin (STZ)-treated model with fat-1 mice. STZ-treated fat-1 mice did not develop hyperglycemia, motor deficits, or Purkinje cell loss. The expression of LC3 I, II, Beclin-1 and p62 were increased in the cerebellum of STZ-treated wild-type mice, and these expressions were more increased in STZ-treated fat-1 mice, but not of p62. Moreover, cerebellar Rab7, Cathepsin D, and ATP6E were increased in STZ-treated fat-1 mice. There was also increased BDNF expression in Purkinje cells without any changes in TrkB, and phosphorylation of Akt and CREB in the cerebellums of fat-1 mice. Collectively, these findings indicate that STZ-treated fat-1 mice were protected from Purkinje cell loss and exhibited increased BDNF signaling, enhancing autophagic flux activity in cerebellar Purkinje neurons. These processes may underlie Purkinje cell survival and may be potential therapeutic targets for treatment of motor deficits related to diabetic neuropathy.

Lipid mediators derived from omega-6 (ω6) and omega-3 (ω3) polyunsaturated fatty acids (PUFA) are important regulators of inflammation and may play key roles in the pathogenesis of diabetes^1^. Studies of Inuit populations in Greenland, whose diets are high in ω3-PUFA from cold water fish oils, have found extremely low incidences of cardiovascular disease, but higher prevalence of type 2 diabetes^2^. Despite these findings, it do not support the idea that fish, seafood, docosahexaenoic acid (DHA), or eicosapentaenoic acid (EPA) affect the development of diabetes mellitus^1^. While ω3-PUFA may have unfavorable effects on type 2 diabetes in Caucasians, they may have beneficial cardioprotective effects, reduce the risk of ischemic stroke in both men and women, and increase insulin sensitivity in Asians^3^. Epidemiological studies have further confirmed that fish-based dietary interventions improve endothelial function in post-menopausal women with type 2 diabetes^4^.

Fat-1 transgenic mice express a Caenorhabditis elegans ω3-desaturase (fat-1), leading to endogenous synthesis of ω3-PUFA from ω6-PUFA. These mice, thus, have higher tissue ω3-PUFA content^5^ and exhibit a more robust anti-inflammatory response in models of mucosal organ injury, including acute lung injury, chemically-induced colitis, hepatitis, and pancreatitis^6^. Resolvins and protectins, the oxygenated products of ω3-PUFA enzymatic metabolism, exert powerful anti-inflammatory and immune-regulatory actions via peroxisome proliferator activated receptors and G-protein-coupled receptor^7^. D-series protectins and resolvins are formed from DHA, whereas E-series resolvins are derived from EPA. Resolvin D1 and resolvin E1 have been previously shown to have potent anti-inflammatory and pro-resolving effects in type 2 diabetes^8^,^9^,^10^.

While diabetic neuropathy has long been considered a disease of the peripheral nervous system, there is increasing evidence that diabetic insult can occur in the CNS, since craniovascular disease appears to be associated with cognitive decline and brain atrophy^11^. Typical symptoms of diabetic neuropathy include pain, numbness, tingling, weakness, and difficulty with balance^12^. Human studies have implicated involvement of the cerebellum in cognitive processing and sensory discrimination in many conditions, such as pervasive developmental disorders, autism, and cerebellar vascular injury^13^,^14^,^15^. There are recent reports that diabetes-induced fusion of adult Purkinje cells with bone marrow-derived cells in the cerebellum leads to the emergence of a sub-population of cells with high pro-inflammatory capacity^16^,^17^. These cells would thereby increase the susceptibility of Purkinje cells to diabetic insult. Consistent with these findings are reports of disruption of cerebellar structure in type 1 diabetes^13^. Streptozotocin (STZ)-induced diabetes in adult rats can also increase apoptosis in cerebellar Purkinje cells and cortical pyramidal neurons^18^. Thus, cerebellar Purkinje cells may play a role in the pathogenesis of diabetic neuropathy. In this study, we used fat-1 transgenic mice to determine the effect of increased DHA and EPA on the development of Purkinje cell degeneration and autophagic dysfunction in STZ-treated mouse model.

## Results

### Effects of endogenous ω3-PUFA in STZ-induced diabetes

**Figure 1:**
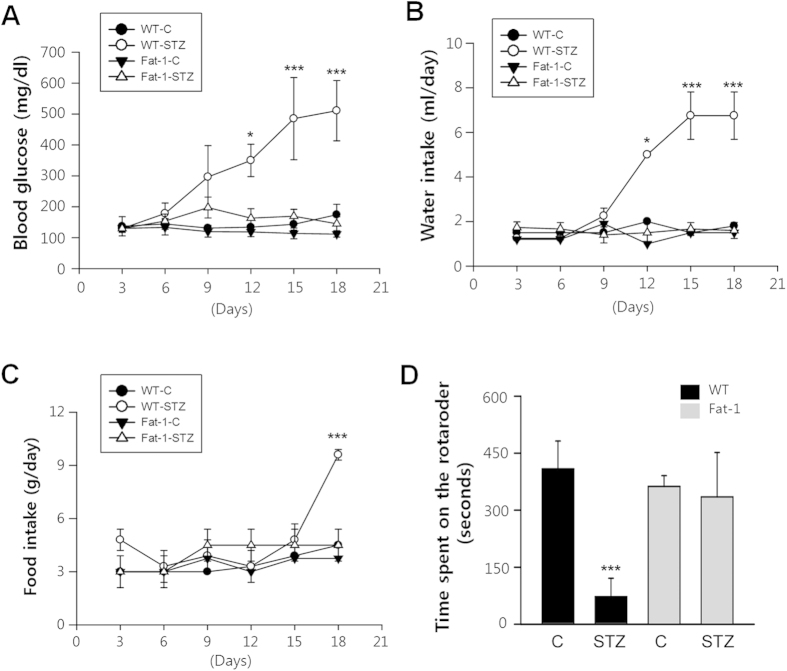
The blood glucose level, intake of water and food, and motor coordination function in STZ-treated fat-1trangenic mice. The blood glucose concentration (**A**), water intake (**B**) and food intake (**C**) of the STZ-treated mice (n = 20/each group) compared with control mice (n = 10/each group) maintained for 18 days of observation. (One-way ANOVA with post hoc Newman-Keuls test, *P < 0.05, ***P < 0.001, vs. control). Error bars indicate mean ± S.D. (**D**) Behavioral analysis of 18 days STZ-treated fat-1 transgenic mice (n = 9/group) and control mice (n = 7/group) in rotarod test. STZ-treated and control mice were test for three consecutive trials on the rotarod. Results were expressed as the time spent (in seconds) on the rotarod.

To determine the effects of fat-1 on diabetic progression, blood glucose concentration and water and food intake were measured in STZ-treated mice. One week after the last injection of STZ, wild-type mice began to develop hyperglycemia, which persisted for the entire 18-day observation period, showing symptoms characteristic of diabetes. In contrast, blood glucose levels in STZ-treated fat-1 mice did not change and were identical to that of citrate-treated wild-type and fat-1 control mice (Fig. 1A). In STZ-treated wild-type mice, mean blood glucose concentration was 510 mg/dl whereas in STZ-treated fat-1 mice and citrate-treated mice, it was 150 mg/dl.

Similarly, food consumption and water intake also increased in diabetic wild-type animals, which exhibited polyphagia and polydipsia. However, food and water intake remained unchanged in STZ-treated fat-1 mice (Fig. 1B,C). Motor coordination was assessed using the total length of time animals spent on a rotating rod. In this test, at 18 days after treatment, STZ-treated fat-1 mice had better motor ability compared with STZ-treated wild-type mice (Fig. 1D). These results suggest that STZ-treated fat-1 mice do not develop hyperglycemia or motor deficits indicative of diabetic neuropathy.

### Effects of endogenous ω3-PUFA on Purkinje cell loss

**Figure 2:**
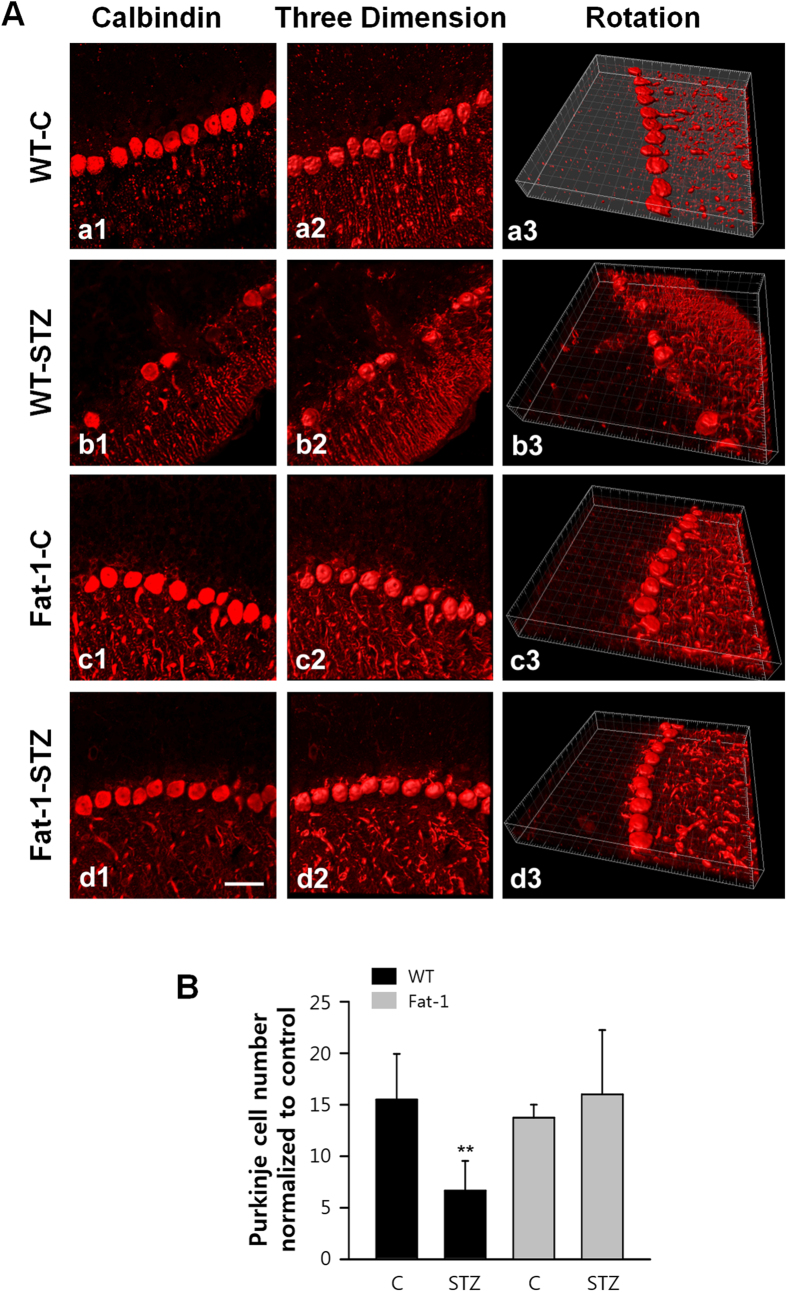
Fat-1 transgene protects animals from STZ-induced Purkinje cell loss. (**A**) Representative confocal images showed the distribution of Calbindin immunoreactive cells by using 3D reconstruction in the cerebellar Purkinje cell layer of wild-type (a1-3, b1-3) and fat-1 transgenic mice (c1-3, d1-3), which obtained at day 18 after the fifth STZ injection. Scale bar = 10 μm. (B) Quantification of Purkinje cell loss of wild-type (n = 7/group) and fat-1 mice (n = 9/group) presented as number of Purkinje cells with same area. Differences were analyzed by the one-way ANOVA with post hoc Newman-Keuls test. ***P < 0.001, vs. control. Data present mean ± S.D.

It was observed a severe cell loss of cerebellar Purkinje cells in STZ-treated wild-type mice by using immunostaining with calbindin, a marker of Purkinje cell (Fig. 2A, b1–3), that is consistent with previous reports^19^,^20^. While treatment with STZ caused severe degeneration and loss in wild-type mice, there were no histological changes in STZ-treated fat-1 mice (Fig. 2A, d1–3). The Purkinje cell number analysis in STZ-treated wild-type and fat-1 mice further confirmed the pattern indicated by immunostaining (Fig. 2B). These results suggest that ω3 enrichment may protect fat-1 transgenic mice from STZ-induced Purkinje cell loss.

### Effect of endogenous ω3-PUFAs on Autophagy flux in Purkinje cell

**Figure 3:**
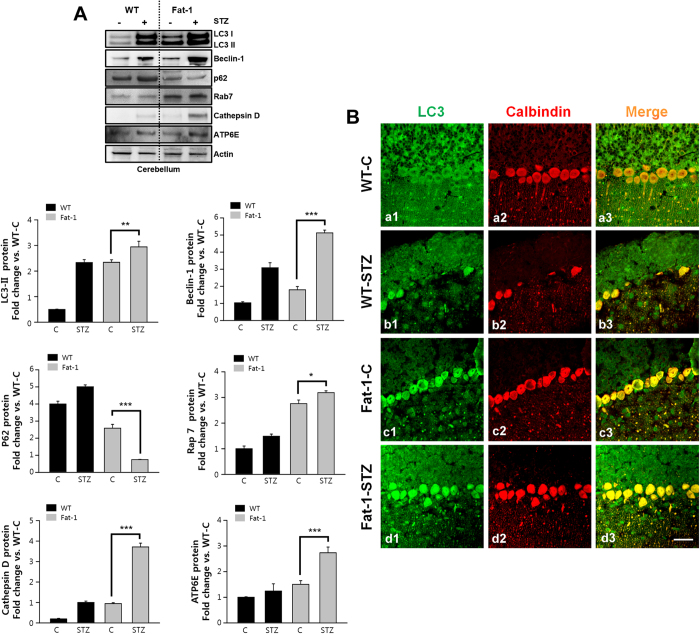
Fat-1 transgene enhances autophagic activity protein expression in Purkinje cells of STZ-diabetic mice. (**A**) Autophagy-associated protein Rab7, LC3I, LC3II, Beclin-1, and p62 levels were analyzed by immunoblotting at day 18 after the fifth STZ injection into wild-type (n = 7/group) and fat-1 transgenic mice (n = 9/group). Differences were analyzed by the one-way ANOVA with post hoc Newman-Keuls test. *P < 0.05, **P < 0.01, **P < 0.001, vs. control. Data present mean ± S.D. of three independent experiments. (B) Representative confocal images showed the LC3 immunoreactivity was colocalized with Calbindin, a marker of Purkinje cells. Scale bar = 10 μm.

It has been hypothesized that the progressive autophagic dysfunction can stimulate apoptosis and degeneration in Purkinje cells of STZ-induced diabetic animals^19^. Therefore, at 18 days after STZ treatment, we investigated the effect of ω3 enrichment on the expression of autophagy-associated protein in Purkinje cells of fat-1 mice. There was elevated microtubule-associated protein 1A/1B-light chain (LC3) I and LC3II in the cerebellum of STZ-treated wild-type mice compared with control, suggesting increase of autophagosomes formation (Fig. 3A, left upper panel). This increase of autophagosomes formation was confirmed by Beclin-1, a well-known key regulator of autophagy. And then, we examined the expression of Sequestosome 1 (SQSTM1/p62) p62 associated with autophagic flux. p62 is involved in autophagy-dependent elimination of many different cargos including ubiqutinated protein aggregates and bacteria. Because of its interaction with LC3, p62 is constantly degraded via autophagy. In other words, autophagy inhibition leads to the accumulation of p62 positive aggregates^21^. The accumulation of p62 observed after STZ treatment suggest that STZ blocks autophagic flux.

In contrast, LC3II and Beclin-1 expression increased in STZ-treated fat-1 mice compared with citrate- and STZ-treated wild-type mice, indicating increase of basal autophagy induction. There was also a reduction in p62 in STZ-treated fat-1 mice compared with citrate-treated wild-type or fat-1 mice (Fig. 3A, right panel). These results, with higher levels of LC3II, Beclin-1 and lower levels of p62, suggest that ω3 enrichment enhances the induction and flux of autophagy.

Moreover, the protein expression of the most substantial molecules in the maturation of autophagosomes/endosomes, such as Rab7, Cathepsin D, and ATP6E were evaluated by immunoblotting. Their levels were found increased in the cerebellum of STZ-treated fat-1 mice compared with control (Fig. 3A, lower panel). We next examined induction of autophagy in Purkinje cells using fluorescence intensity of LC3 (the total form of LC3I and II). The expression of LC3 was increased in Purkinje cells of cerebellum than other layers, such as molecular, granular layer and white matter (Fig. 3B). These results suggest that autophagic impairment, such as autophagic flux blocks and autophagic maturation impairment was found in the Purkinje cells of STZ-treated control and fat-1 mice,

### Effects of endogenous ω3-PUFA on BDNF and downstream signaling

Autophagy has recently been associated with brain-derived neurotrophic factor (BDNF)^22^, a member of the mammalian neurotrophic family, which has been shown to be a potent growth factor beneficial for neuronal function. BDNF exerts its effects by binding its receptors, tyrosine kinase B (TrkB) and p75^23^,^24^. Mature BDNF triggers three intracellular signaling cascades, the MAPK, PI3K, and PLCγ pathways, which are its predominant downstream effectors^25^.

**Figure 4:**
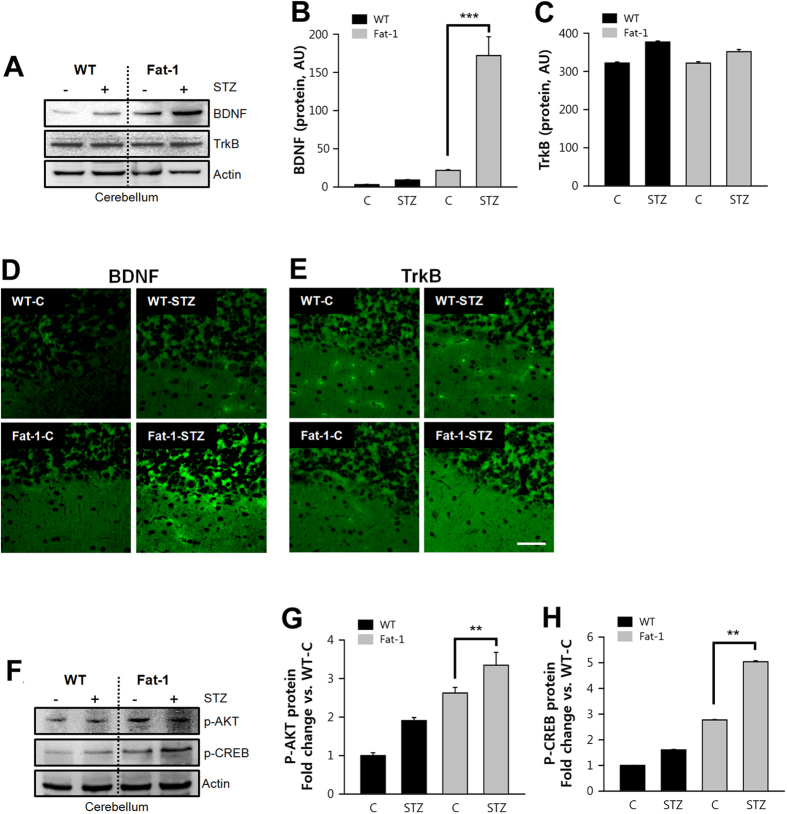
Increased BDNF in fat-1 transgenic mice enhanced Purkinje cell survival after STZ-treated animal model. (**A**–**C**) BDNF and its receptor, TrkB protein levels were analyzed by immunoblotting at day 18 after the fifth STZ injection into wild-type (n = 7/group) and fat-1 transgenic mice (n = 9/group). The ratio of BDNF and TrkB vs. actin were shown in the right panel (one-way ANOVA with post hoc Newman-Keuls test, ***P < 0.001, vs. control. Data present mean ± S.D. of three independent experiments). (**D**) Representative confocal images showed the BDNF immunoreactivity was found in Purkinje cells of STZ-induced fat-1 transgenic mice, but TrkB was hardly detected. Scale bar = 10 μm. (**F**–**H**). BDNF and TrkB receptor-associated downstream signaling molecules, Akt and CREB protein levels were analyzed by immunoblotting in STZ-induced wild-type (n = 7/group) and fat-1 transgenic mice (n = 9/group). The ratio of phospho-Akt and phospho-CREB vs. actin were shown in the right panel (one-way ANOVA with post hoc Newman-Keuls test, **P < 0.01, vs. control. Data present mean ± S.D. of three independent experiments).

In this study, there were higher levels of BDNF in the cerebellums of fat-1 mice, as determined by immunoblotting, while TrkB remained unchanged (Fig. 4A–C). These results were corroborated by increased immunofluorescence intensity of BDNF, but not TrkB, in Purkinje cells (Fig. 4D,E). The trophic effects of BDNF binding to TrkB are due to activation of various signaling cascades, including the extracellular signal-regulated kinase (ERK) and PI3K pathways. In this regard, the PI3K/Akt/mTOR/p70S6K signaling pathway, in particular, is deemed to be important in regulating autophagy^22^,^26^,^27^. Since higher levels of BDNF were found in Purkinje cells of fat-1 mice, we monitored regulation of TrkB signaling using phospho-Akt. Thr308 phosphorylation of Akt was increased in fat-1 mice compared with control (Fig. 4F,G).

To further evaluate the effects of Akt phosphorylation, we monitored Ser133 phosphorylation of cAMP response element-binding protein (CREB) since CREB is a regulatory target for the protein kinase Akt^28^. CREB activation in neurons can stimulate the expression of neuroprotective molecules, such as anti-apoptotic protein and Bcl-2, which contribute to the survival of cells after ischemic or neurotoxic insult^29^,^30^. Ser133 phosphorylation of CREB was increased in the cerebellum of fat-1 mice compared with control (Fig. 4H). Collectively, these data support the idea that ω3 enrichment can stimulate BDNF expression, leading to activate Akt and CREB by increasing Thr308 phosphorylation of Akt and Thr133 phosphorylation of CREB (Fig. 4G,H).

## Discussion

To the best of our knowledge, this study demonstrates for the first time that BDNF expression increases in fat-1 transgenic mice. We suggest that the increased level of BDNF may protect Purkinje cells from STZ-induced injury *in vivo* via enhanced autophagic flux and activation of the PI3K/CREB signaling pathway.

Diabetic neuropathy is the most common complication and the most significant cause of morbidity and mortality in diabetic patients. There is now increasing evidence of CNS involvement in diabetic neuropathy^31^. It has recently been reported that STZ-treated rats exhibit dysfunction in motor coordination, degeneration and loss of Purkinje cells, and impaired autophagy, as evidenced by lack of autophagosome formation and aggregation of p62 in Purkinje cells^19^. Our present findings in Purkinje neurons of STZ-treated mice are consistent with this previous report. In addition, our results show that endogenous production of ω3-PUFAs in fat-1 mice prevents the development of hyperglycemia and Purkinje cell loss. We propose that there is a relationship between ω3-PUFA levels and protection from hyperglycemia.

The formation of functional neural systems requires massive cytoplasmic remodeling that may involve autophagy, an important intracellular mechanism for the degradation of damaged proteins and organelles. For example, lack of Atg7 in pro-opiomelanocortin (POMC) neurons, a major negative regulator of energy balance, causes higher post-weaning body weight, increased adiposity, and glucose intolerance^32^. These metabolic impairments were associated with an age-dependent accumulation of ubiquitin/p62-positive aggregates in the hypothalamus and a disruption in the maturation of POMC-containing axonal projections^32^. These findings suggest that autophagic dysfunction might have harmful effects on neurons and that appropriate autophagic activity is required for normal metabolic regulation. It is well-known that autophagy plays a major role in the peripheral regulation of metabolism, affecting pancreatic, liver, and adipocyte morphology and function^33^,^34^,^35^. Mice deficient in either Atg5 or Atg7 in the CNS develop progressive behavioral and motor deficits typically associated with neurodegenerative diseases. Consistent with these observations, the same mutant mice display early signs of neurodegeneration, including extensive neuronal loss and abnormal protein aggregation in the cortex and the cerebellum^36^,^37^. Thus, Purkinje cells may be vulnerable to both STZ-treated hyperglycemia and perturbation of autophagic function. In this study, we found higher levels of lipidated LC3II and Beclin-1 and lower levels of p62 in the Purkinje cells of fat-1 transgenic mice.

In addition, Rab7, Cathepsin D and ATP6E, which play a role in maturation of autophagolysomes, were also elevated in fat-1 transgenic mice. Rab7, a member of small GTPases, designates the maturation of endosomes and also autophagosomes, directing the trafficking of cargos along microtubules, and finally, participating in the fusion step with lysosomes^38^. Cathepsin D is an aspartic proteinase and a lysosomal enzyme digesting molecules and organella in autolysosomes. Cathepsin D plays an important role in protein degradation, particular in CNS tissues^39^. ATP6E, also known as V-ATPase E, is a vacuolar-type H+-ATPase (V-ATPase). ATP6E controls acidification of the vacuolar system and provides the main proton-motive force^40^. Lysosomal acidification is crucial for the degradation of engulfed materials and is an important marker of functional lysosome. All proteins play an important roles in lysosome/autophagosomes fusion processes and finally are associated with autophagic flux^41^. Taken together, these data suggest that enrichment of ω3-PUFA might counteract autophagic dysfunction due to STZ-treated hyperglycemia. Preservation of autophagic function might thereby prevent Purkinje cell loss and subsequent development of motor deficits.

BDNF exerts its effects through high-affinity binding with its receptor, TrkB. BDNF and TrkB are distributed in sub-regions of the hippocampus, forebrain, and cerebellum^42^,^43^. It is known to be involved in the regulation of synaptic function and synaptic plasticity. BDNF has also been suggested to play a critical role in the development, differentiation, and protection of retinal neurons^44^. Administration of exogenous BDNF protects retinal ganglion cells during optic nerve axotomy^45^, retinal ischemia^46^, and NMDA-induced neuronal death *in vivo*^47^. Few studies have investigated the role of autophagy in BDNF-mediated protection against diabetes. However, it has been recently reported that the neuroprotective effect of BDNF is mediated by autophagy through the PI3K/Akt/mTOR pathway in cortical neurons^22^. In those studies, the authors show that autophagy was induced in oxygen-deprived cells, and that BDNF promoted cell viability via up-regulation of autophagy. Moreover, they suggested that LC3 up-regulation is related to Akt/mTOR/p70S6K pathways by BDNF. These results suggest that BDNF-induced autophagy might contribute to protection of cortical neurons during hypoxia. In line with those findings, we demonstrate that BDNF was expressed at higher levels in the Purkinje cells of fat-1 mice, and that BDNF-associated proteins, such as Akt and CREB, were elevated in the cerebellum.

Autophagy is an essential mechanism for maintaining cellular homeostasis during stress conditions, such as diabetic neuropathy. Our results suggest that autophagic activity in fat-1 transgenic mice might be important to defense mechanisms activated by diabetic neuropathy *in vivo*. BDNF might be a novel neuroprotective factor, in this respect, for its potential up-regulation of autophagic activity, which presumably prevented Purkinje cell loss. Further studies are required to confirm the specific effects and mechanisms of BDNF.

## Materials and Methods

### Materials

Streptozotocin (STZ, S0130), LC3 (for immunoblotting, #L8918), and p62 (#P0067) were purchased from Sigma (St. Louis, MO, USA). LC3 for immunostaining (#PM036) was purchased from MBL international (Woburn, MA, USA). Cathepsin D (sc-377124), ATP6E (sc-20946) and Beclin-1 (sc-11427) were purchased from Santa Cruz Biotechnology (Santa Cruz, CA, USA). p-AKT (#2965), Rab7 (#9367) and beta-actin (#4970) were procured from Cell signaling (Danvers, MA, USA). Phospho-CREB (#05-807) and TrkB (#07-225) were purchased from Millipore (Billerica, MA, USA). BDNF (AB1779SP) and calbindin (AB1778) were purchased from Chemicon (Billerica, MA, USA).

### Animal model

Dr. J.X. Kang at the Harvard Medical School (Boston, MA, USA) kindly provided the fat-1 transgenic mice. Male mice were housed individually in cages on a standard 12:12 h light:dark cycle. Water and food were available ad libitum until mice were transported to the laboratory, approximately 1h prior to experiments. Presence of the fat-1 gene was confirmed by genotyping. All experiments were carried out with the approval of the Animal Care and Use Committee at KonYang University and were consistent with the ethical guidelines of the National Institutes of Health and the International Association.

### STZ administration

Diabetes was induced using STZ, as previously described^48^. Briefly, STZ (2-deoxy-2–3-[methyl-3-nitrosoureido]-D-glucopyranose; Sigma, St. Louis, MO) was dissolved in 0.1 M sodium citrate buffer (pH 4.5) and injected intraperitoneally within 15 min of preparation, at a dose of 50 mg/kg/day for 5 consecutive days to produce a beta cell destruction model. Control wild-type and transgenic mice were injected with the citrate buffer vehicle. Blood glucose level was measured in non-fasted animals from tail venous blood using a glucometer (LifeScan, Milpitas, CA). Mice were evaluated every 2 days at 2:00 P.M. and were considered diabetic when blood glucose levels exceeded 250 mg/dl, usually 7 to 9 days after the last STZ injection. All mice were sacrificed by a blind investigator for tissue collection.

### Behavioral testing

Motor coordination and balance were evaluated using the rotarod test. We performed the test using an accelerating rotarod by placing a mouse on a rotating drum (2.5 cm diameter) and measuring the time during which the animal was able to maintain its balance on the rod (latency time to fall in seconds). During training, mice were placed on a rotarod accelerated from 4 to 40 rpm. over 300 seconds. Animals underwent repeated testing for a total of three times.

### Immunohistochemical and confocal microscopic analysis

Formalin-fixed, paraffin-embedded sections of cerebellum were stained with calbindin, LC3, BDNF, and TrkB. Sections were de-paraffinized and dehydrated in a graded series of ethanol washes. Tissue sections were subjected to heat-induced epitope retrieval (0.1 M citrate buffer), then cooled at room temperature for 20 min. After washing in phosphate-buffered saline (PBS) (pH 7.4), endogenous peroxidase was quenched using a 3% solution (v/v) of hydrogen peroxidase and PBS. After blocking non-specific binding with 1.5% BSA in PBS, sections were incubated with primary antibodies overnight at 4 °C. Sections were then incubated with a secondary antibody, washed again in PBS, and then incubated for 20 min with horseradish peroxidase-3-amino-9-ethylcarbazole detection solution. After mounting, fluorescence images were acquired using a confocal laser-scanning microscope (LSM 700; Zeiss, Thomwood, NY, USA). Serial optical sections at intervals of 1.5 μm in the z dimension were captured to allow for three-dimensional (3D) reconstruction. Imaris 7.1.1 (Bitplane), Ultraview 5.5 (PerkinElmer), and Adobe Photoshop 7 (Adobe Systems) were used for image processing.

### Western blot

Cerebellar tissue from STZ-treated and control mice was separated by electrophoresis before being transferred to polyvinylidene fluoride (PVDF) membranes. Total protein extracts were obtained by homogenization in a protein lysis buffer supplemented with a mixture of protease inhibitors (Complete; Roche Applied Science, Upper Bavaria, Germany). Protein extracts were fractionated on a proper concentration of SDS-PAGE and then transferred onto PVDF membranes. The membranes were blocked with blocking buffer containing 5% non-fat dry milk for 2 h at room temperature and then incubated with rabbit primary antibodies (anti-Rab7, anti-LC3, anti-Beclin-1, anti-p62, anti-BDNF, anti-TrkB, anti-phospho-AKT, anti-phospho-CREB, anti-actin, as appropriate) at 4 °C overnight. The membranes were washed three times with tris-buffered saline with 0.1% Tween 20, for 10 min, and then incubated with HRP-conjugated anti-rabbit IgG secondary antibodies (1:1000) for 2 h at room temperature. Enhanced chemiluminescence was used for detection.

### Statistical analysis

Data obtained from independent experiments (mean ± SD) were analyzed using two-tailed a Student’s t-test. Differences were considered significant if P < 0.05. 95% confidence intervals were computed, 1.96 × standard error in each direction. Statistical significance was evaluated using a log-rank (Mantel-Cox) test.

## Additional Information

**How to cite this article**: Bak, D. H. *et al.* High ω3-polyunsaturated fatty acids in fat-1 mice prevent streptozotocin-induced Purkinje cell degeneration through BDNF-mediated autophagy. *Sci. Rep.* **5**, 15465; doi: 10.1038/srep15465 (2015).
